# Investigating performance and key factors for real-world deployment of grain image classification using convolutional neural networks

**DOI:** 10.1038/s41598-026-45314-6

**Published:** 2026-04-14

**Authors:** Rani Kumari, Jonas Persson, Veronica Ehrström Eklöf, Moa Källgren, Ida-Maria Sintorn

**Affiliations:** 1 Department of Information and Technology, Uppsala University, Uppsala, Sweden; 2 CGrain AB, Uppsala, Sweden

**Keywords:** Image classification, Class imbalance, Wheat grain quality assessment, Deep learning, Convolutional neural networks (CNN), Uniform manifold approximation and projection (UMAP), Engineering, Mathematics and computing

## Abstract

Accurate and efficient grain quality assessment is critical for making informed decisions throughout the grain value chain. Early detection of disease enables actions to mitigate spread and further damage, and optimal batch mixing to fulfill specified quality requirements allows for maximizing value and minimizing scrapping. Vision based machine learning and deep learning approaches are gaining attention in the agricultural sector and are useful for the development of automated grain quality assessment. These techniques can reduce the current manual inspection load and are key for objective and precise analysis. Yet, the majority of prior studies are constrained to small or controlled and curated datasets. Practical challenges associated with real-world deployment and reliability are rarely addressed. That is the focus of this work. We present and demonstrate a structured approach for investigating convolutional neural networks (CNNs) and key factors influencing performance for wheat kernel classification. The objective is to determine a CNN model that ensures high and robust classification accuracy, while elucidating and explaining how different image dataset characteristics and training parameters affect performance and reliability. We use a commercial mirror-based imaging system that captures over 90% of each kernel’s surface and contrast and compare model architectures, robustness, the effect on pre-processing and image resolution. Our results show similar and high overall performance for ResNet50V2 and EfficientNetV2B0 ($>96$% accuracy), but per-class analysis indicate that the smaller classes suffer from lack of representative examples, and that most classes benefit from pre-processing including downsampling whereas others benefit from higher resolution. Interactive visualizations reveal that another contributing factor is dubious annotation and multi-class belongingness. Thus, our step-by-step analysis of CNN performance underscores the need for representative data, proper pre-processing, and class-aware evaluation to ensure trustworthy deployment in wheat grain quality assessment.

## Introduction

Wheat (*Triticum aestivum*) is a staple food crop and one of the most widely cultivated cereals globally, with a production volume exceeding 791 million tonnes in 2023–24 while growing with 0.14% annually^1^. As the global population is projected to reach 9.7 billion by 2050^2^, researchers and agricultural experts are striving to improve wheat yields to meet the growing food demand. In addition to yield improvements, the quality of wheat grains plays a pivotal role in ensuring food security, fair market pricing, and efficient supply chain management. Accurate and high-throughput methods for grain quality assessment are critical for achieving these goals.

**Fig. 1:**
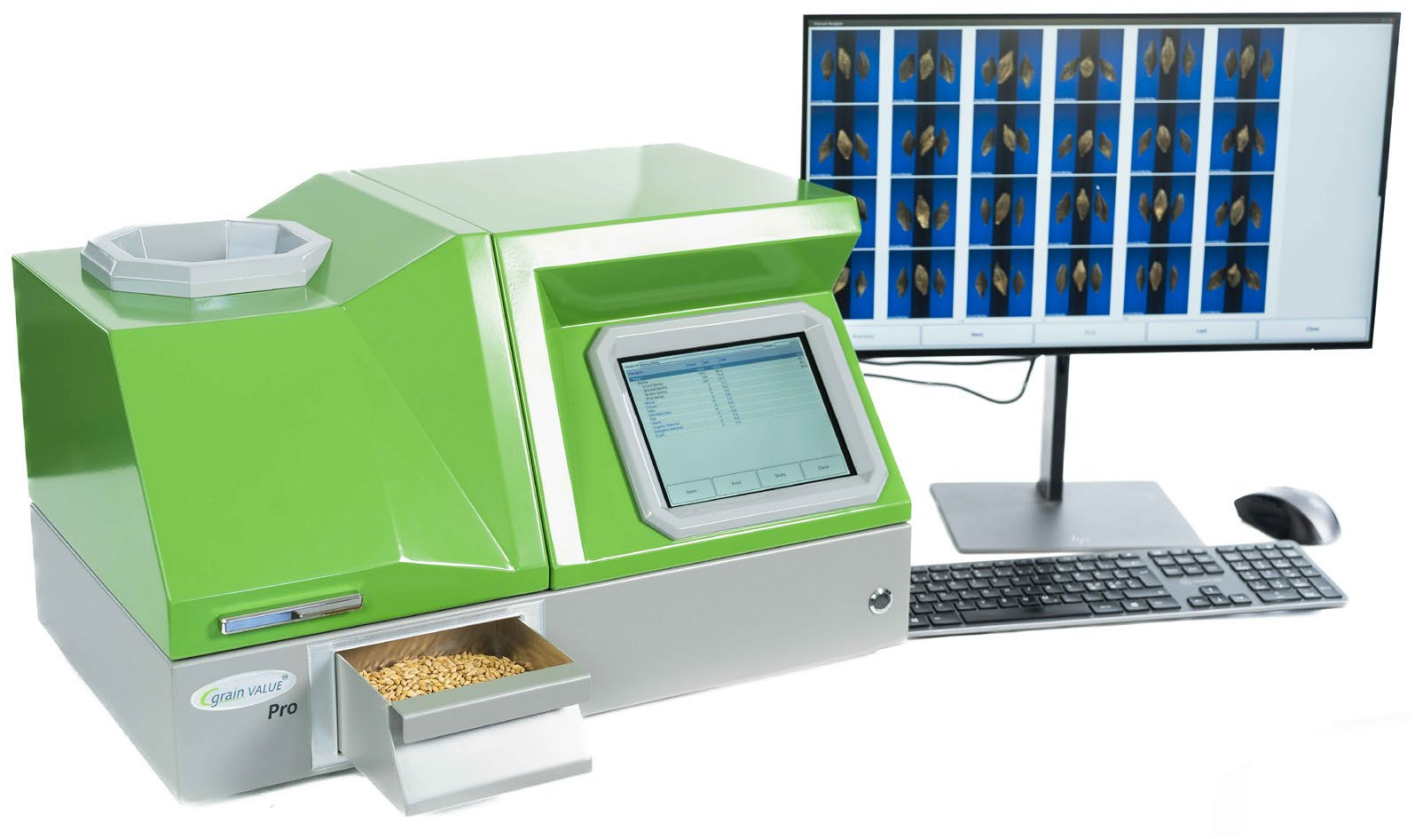
The Cgrain Value Pro grain kernel analysis system^5^.

Traditional wheat grain classification has predominantly relied on manual inspection^3^, which, although performed by skilled professionals, is labor-intensive, subjective, and error-prone^4^. To address these limitations, the CGrain$^2$ imaging system, shown in Fig. 1, employs a mirror-based setup, high-resolution cameras, and LED flash lighting to capture detailed images of individual wheat kernels. These images are then automatically analyzed using a combination of conventional and deep learning image processing techniques to extract quality characteristic measures^5^. Numerous studies have investigated automated grain classification using methods like clustering, thresholding, and traditional machine learning models such as Support Vector Machines (SVMs)^6,7^ These approaches typically rely on handcrafted features, including grain size, shape, and color. CNNs for grain classification^8–12^ can automatically extract features from images, eliminating the need for manual feature engineering while generally improving classification accuracy. Researchers have successfully applied such CNN models to various crop production tasks, for example disease detection in wheat^13^, yield estimation from satellite images^14^, and rice grain phenotyping^15^.

While CNNs have demonstrated strong potential in these agricultural applications, in challenges such as agriculture product monitoring^16,17^, computer vision still faces several problems such as class imbalance, variations in imaging conditions, and the need to retain fine-grained details. Careful consideration of these factors is essential to achieve reliable and good real-world classification systems. We examine the impact of CNN architecture choices, data split strategies, and pre-processing methods using wheat kernel images at varying resolutions to achieve this. The primary objectives of this study are:

- Investigate how different CNN architectures affect the classification accuracy.
- Assess the effect of data splits and random seeds to gain insights into robustness and generalization.
- Evaluate the influence of resolution and image pre-processing on classification performance.

Our motivation is that by gaining insights into whether and how these factors influence performance, informed decisions about real-world use and the extent to which the system can be trusted can be made. Moreover, identifying weak links enables the design and development of improved classification systems.

The remainder of this paper is structured as follows: Section “Related work” reviews previous research on conventional and deep learning-based approaches for wheat grain classification. Section “Data and CNN architectures” details the dataset and class distribution. It also outlines the experimental setup, including the selection of CNN architectures and evaluation metrics. Section “Experiments and results” presents the experimental findings, including implementation details, training configurations, and hyperparameters. Section “Discussion” discusses the results and their implications and finally, Section “Conclusion” summarizes key insights and outlines potential directions for further improvements. In addition, we provide additional detailed results and visualizations in “Supplementary Material”.

## Related work

Machine learning and deep learning have advanced image-based grain classification, by providing efficient, scalable, and automated solutions for quality assessment and defect detection, as demonstrated in^8,9,18^ and^19^. Over the years, the transition from conventional machine learning methods to deep learning-based approaches has significantly improved classification accuracy, generalization, and adaptability to real-world agricultural applications.

Agarwal et al.^6^ proposed a machine learning-based system to classify wheat grains as fresh or rotten in bulk images. They used handcrafted features and their classification relied on SVMs, achieving an accuracy of 93%. Velesaca et al.^8^ provided a comprehensive review of computer vision techniques for food grain classification, emphasizing advances in image acquisition, pre-processing, and classification pipelines. Their study explored hyperspectral and multispectral imaging to address minimal inter-class variability among grain varieties. Similarly, Lingwal et al.^9^ developed a CNN-based wheat variety classification model, reporting an accuracy of 97.53%. However, their work was limited to small datasets.

Recent research has also demonstrated the advantages of integrating spectral imaging with deep learning. Chen et al.^10^ achieved high classification accuracy (98.7% and 97.8% for calibration and prediction) using Terahertz Time-Domain Spectroscopy (THz-TDS) in combination with CNNs. Their study focused on classifying 12 different wheat grain varieties by leveraging THz-TDS to capture spectral data and applying CNNs to effectively differentiate between them. However, they utilized only a single CNN model, the results highlight the potential of combining terahertz spectroscopy with deep learning for accurate and efficient wheat variety identification.

Hybrid models that merge traditional machine learning techniques with deep learning have also demonstrated potential. Unlersen et al.^7^ proposed a hybrid approach utilizing CNNs for feature extraction and SVMs for classification, achieving 98.1% accuracy on bulk wheat samples. Yasar et al.^20^ extended this work by incorporating feature selection techniques and SVMs for wheat seed classification, balancing computational efficiency with high accuracy. Another study by Yasar et al.^11^ introduced a deep learning model that integrates the Xception CNN architecture with Bidirectional Long Short-Term Memory (BiLSTM), reaching 97.73% accuracy in classifying five varieties of bread wheat. While these studies demonstrate the effectiveness of combining deep learning with traditional machine learning methods. They focus on bulk or lower-resolution grain images rather than high-resolution individual kernel classification.

Yasar et al.^12^ evaluated ResNet18, MobileNet, and InceptionV3 for bread wheat variety classification on a large, high-resolution dataset, demonstrating that these models consistently achieved over 97% accuracy. Similarly, Kozłowski et al.^21^ developed a specialized dual-sided imaging system for malting barley kernel classification, leveraging controlled imaging conditions and high-resolution captures to achieve 94% accuracy using a custom deep learning model. These studies highlight the potential of deep learning in grain classification, but their focus is on variety classification using controlled imaging systems and large datasets. That makes their problems relatively easier compared to defect detection, which requires identifying subtle, irregular anomalies.

## Data and CNN architectures

### Data collection

**Fig. 2:**
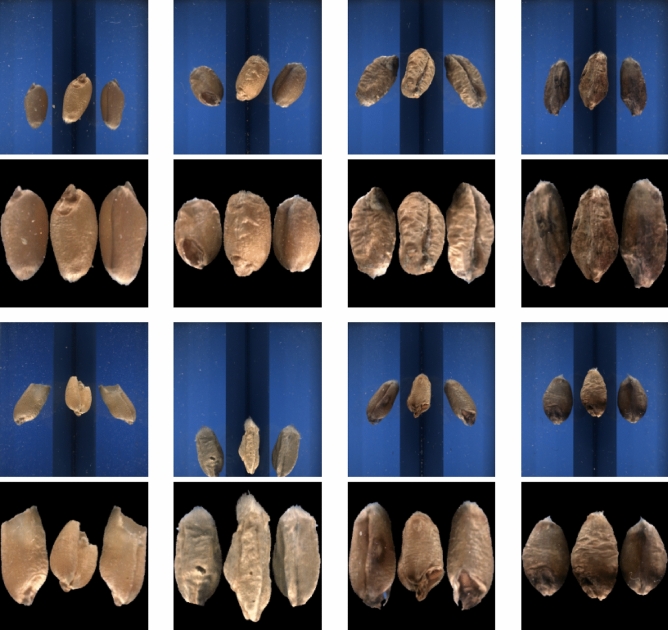
The first row (left to right) shows raw images of: (**a**) Sound, (**b**) Insect, (**c**) Moldy, and (**d**) Spotted kernels. The second row displays their corresponding pre-processed segmented versions. The third row (left to right) presents raw images of: (**e**) Broken, (**f**) Fusarium, (**g**) Sprouted, and (**h**) Black Germ kernels, followed by their segmented versions in the fourth row.

Our dataset consists of 207,658 manually annotated wheat grain images, acquired at seven different time points using two instruments of the *CGrain* automated seed kernel imaging system, shown in Fig. 1. The images are captured as $792 \times 830$ pixel-low compression jpeg RGB images and then processed using Cgrain’s built-in proprietary pre-processing (color correction, segmentation of the three mirror views, alignment, and downscaling) to create $256 \times 256$ segmented images. Each color band is intensity normalized to the range [0,1]. The dataset consists of eight classes: *Sound (healthy kernels), Fusarium (kernels infected by Fusarium fungus), Moldy (general fungal damage), Broken (physically damaged kernels), Spotted (surface discoloration), Sprouted (premature germination), Black Germ (dark embryo infection), and Insect (insect-damaged kernels).* Examples of an original image, as well as a segmented and resampled image from each class, are shown in Fig. 2.

**Fig. 3:**
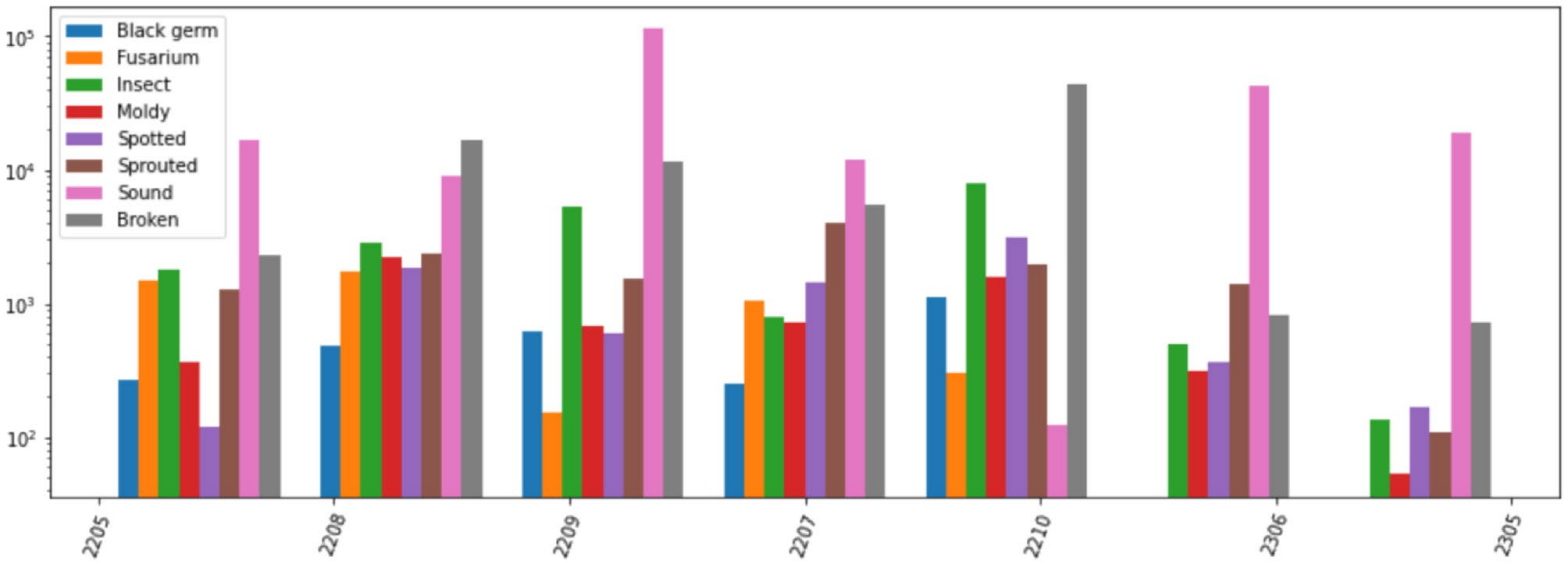
Class distributions at the different time points (year, month) over which the data set was collected..

Figure 3 illustrates the class distribution at the different time points over which the data set was collected. Notably, the dataset exhibits significant class imbalance, with “Sound” comprising nearly half (49.02%) and “Black Germ” representing only 1.08% of the data. The Figure also helps to visualize the differences between Test Set-1 (time point 2208) and Test Set-2 (time point 2207) in terms of class representation. It ensured that the test sets reflect different data distributions, yet contain all classes which is important to analyze generalization performance.

Table 1 presents the detailed proportion of data for each class. These imbalances are natural in real-time scenarios and highlight the need for methods to ensure robust classification across all classes. The dataset was divided into training, validation, and test subsets to evaluate the performance of the different classification models. The images of two sessions (time points) were used one at a time as the test set. The remaining images (including all time points not currently used as test) were split into training and validation sets following a 9: 1 ratio, with images randomly selected from each class.

**Table 1:** Data class distribution.

Class	Training Set	Test Set-1	Test Set-2
Sound	101798	8949	11807
Fusarium	2960	1725	1028
Moldy	3716	2196	710
Broken	64726	16759	7307
Spotted	5845	1827	1422
Sprouted	10037	2351	3865
Black Germ	2245	486	240
Insect	16331	2850	765
Total	207658	37143	27144

### CNN selection and evaluation strategy

Three well-established CNN models: *MobileNetV2*^22^, *EfficientNetV2B0*^23^, and *ResNet50V2*^24^, were selected based on their demonstrated efficiency in image classification tasks.

*MobileNetV2* is a lightweight model (3.4M parameters) designed for mobile and embedded devices. It achieves high efficiency by using depthwise separable convolutions, which reduce both the number of parameters and computational cost. However, while highly efficient, it might not consistently achieve the highest accuracy compared to deeper, more complex models.

*EfficientNetV2B0*, uses a compound scaling method to balance depth, width, and resolution, allowing it to achieve a high level of accuracy while still being computationally efficient. This model is often preferred when the trade-off between accuracy and computational efficiency needs to be carefully balanced. EfficientNetV2B0 is particularly effective for high-performing applications but focuses on reasonable model size (24M parameters) and inference time.

*ResNet50V2* is chosen for its residual learning approach and good performance on complex datasets with hierarchical and subtle features. The deep residual architecture allows the model to train effectively by mitigating the vanishing gradient problem, making it highly effective for tasks that require learning from complex data patterns. Although ResNet50V2 (25.6M parameters) is computationally heavier than MobileNetV2 and EfficientNetV2B0, it is often a good choice for tasks that prioritize accuracy and the ability to capture intricate features.

To assess the model’s performance, we employed standard evaluation metrics: accuracy^25^, balanced accuracy^26^, precision, recall^27^ and F1-Score, see equations (1) to (5), where *TP* and *FP* are the true and false positives, respectively. *FN* are the false negatives, and *C* represents the number of classes. Precision and recall are computed for each class to evaluate the model’s performance in classifying specific categories.

- *Accuracy* Measures the proportion of correct predictions across all classes:
  1 $$\begin{aligned} \text {Accuracy} = \frac{\text {Correct Predictions}}{\text {Total Predictions}} \end{aligned}$$
- *Precision* Evaluates the proportion of true positives to predicted positives for each class:
  2 $$\begin{aligned} \text {Precision}_i = \frac{\text {TP}_i}{\text {TP}_i + \text {FP}_i} \end{aligned}$$
- *Recall* Measures the proportion of true positives to actual positives for each class:
  3 $$\begin{aligned} \text {Recall}_i = \frac{\text {TP}_i}{\text {TP}_i + \text {FN}_i} \end{aligned}$$
- *F1-Score* Represents the harmonic mean of precision and recall for each class:
  4 $$\begin{aligned} \text {F1-Score}_i = 2 \cdot \frac{\text {Precision}_i \cdot \text {Recall}_i}{\text {Precision}_i + \text {Recall}_i} \end{aligned}$$
- *Balanced accuracy* Accounts for class imbalance by averaging recall across all classes:
  5 $$\begin{aligned} \text {Balanced Accuracy} = \frac{1}{C} \sum _{i=1}^C \frac{\text {TP}_i}{\text {TP}_i + \text {FN}_i} \end{aligned}$$

## Experiments and results

We conducted two experiments to analyze how architecture selection, pre-processing techniques, and image resolution affect classification accuracy and generalization of performance.

**Table 2:** Performance metrics across different models and seeds.

Model	Seed	Train Acc (%)	Val Acc (%)	Test Acc (Bal Acc) (%)
MobileNetV2 (Test-set 1)	A	97.0	94.8	94.2 (89.0)
MobileNetV2 (Test-set 1)	B	97.1	84.8	85.0 (86.2)
MobileNetV2 (Test-set 1)	C	96.9	90.5	90.6 (88.4)
EfficientNetV2B0 (Test-set 1)	A	97.1	94.2	96.5 (93.1)
EfficientNetV2B0 (Test-set 1)	B	96.9	94.3	96.2 (92.6)
EfficientNetV2B0 (Test-set 1)	C	96.9	95.4	96.5 (93.0)
ResNet50V2 (Test-set 1)	A	98.2	95.3	96.3 (91.4)
ResNet50V2 (Test-set 1)	B	98.2	93.7	95.1 (91.3)
ResNet50V2 (Test-set 1)	C	98.2	95.2	96.2 (91.9)
ResNet50V2 (Test-set 2)	A	96.9	95.2	90.6 (82.5)

**Fig. 4:**
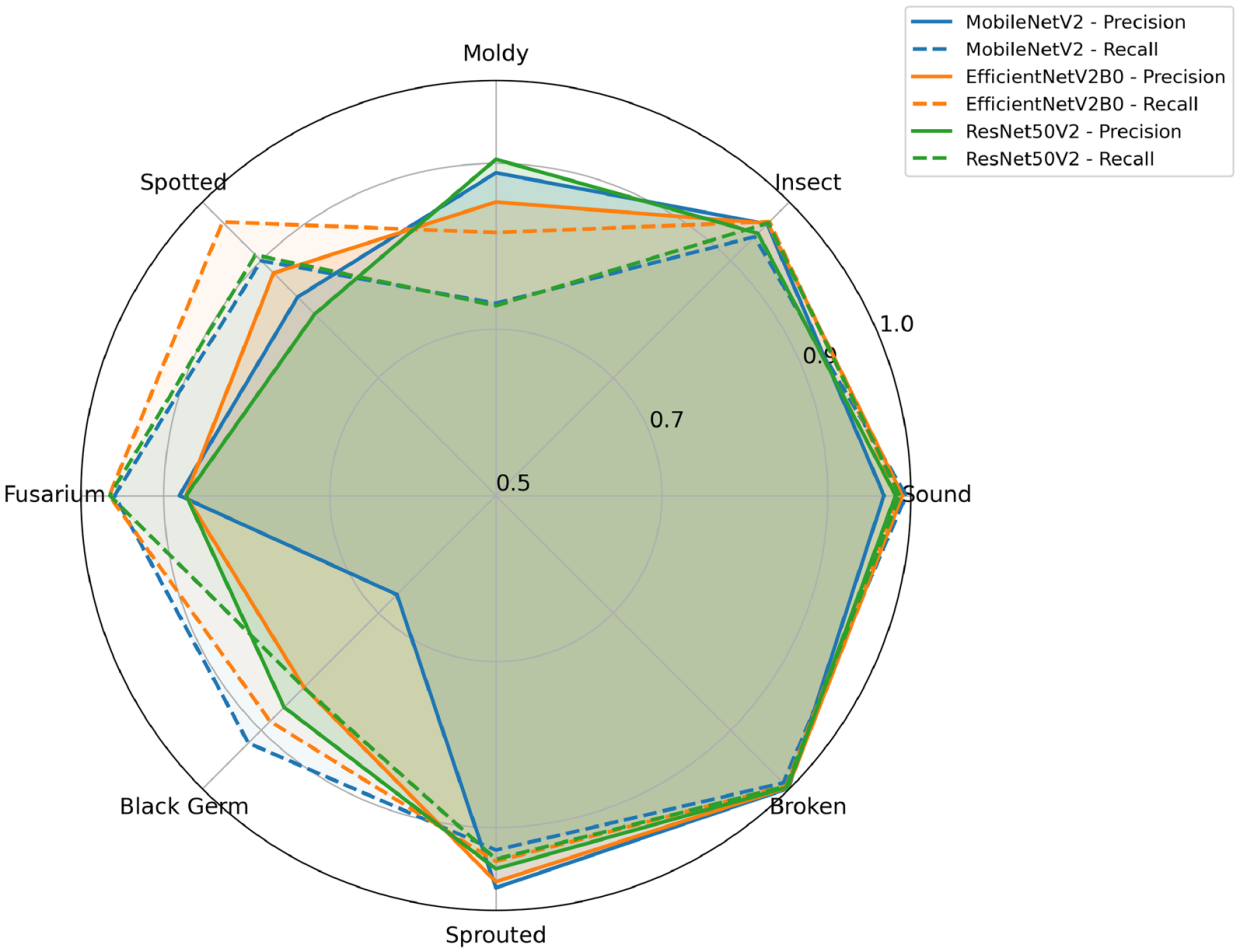
Per-class Precision and Recall for segmented image models (Test Set-1 with Seed A).

**Fig. 5:**
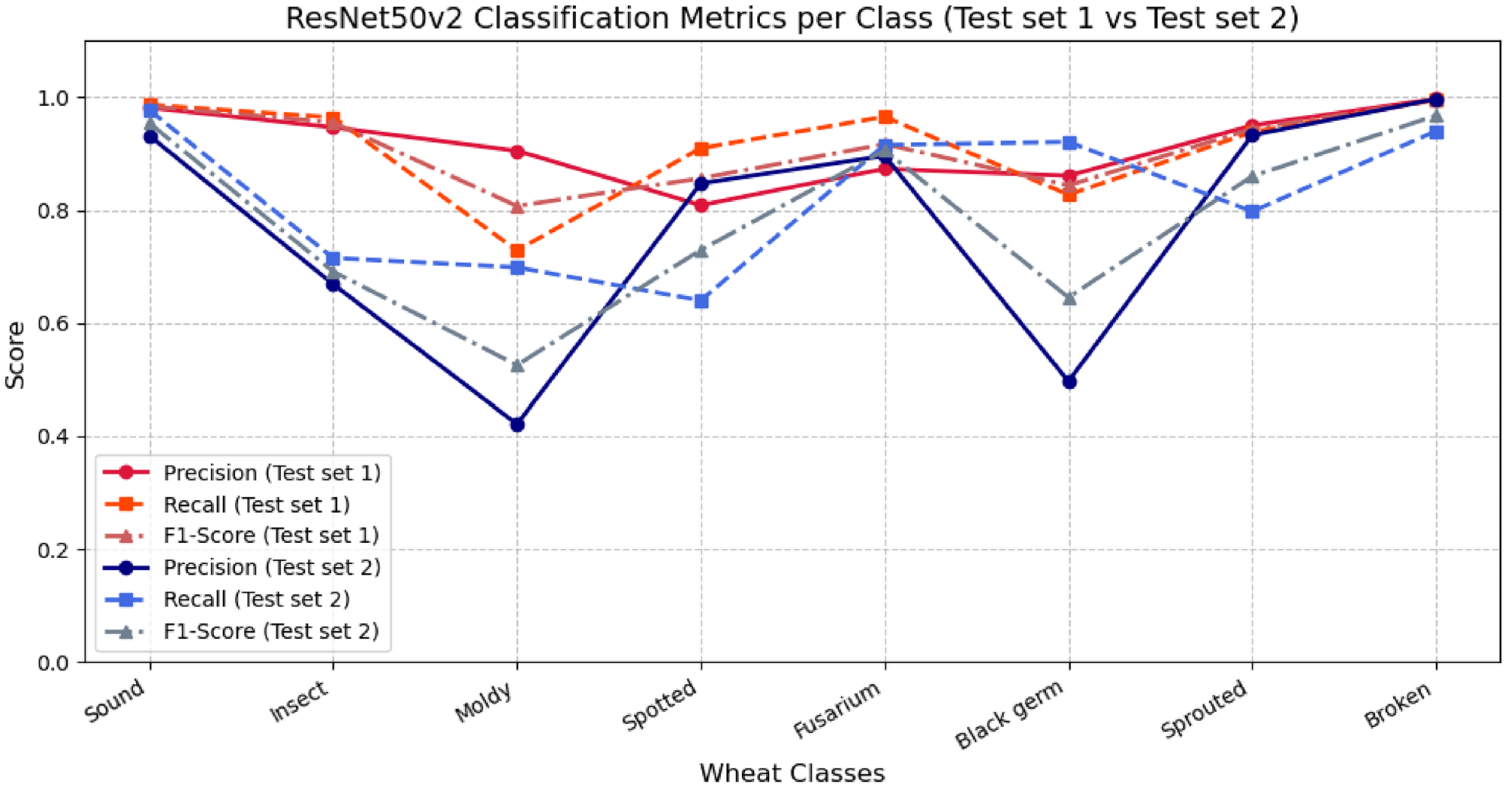
Comparison of per-class metrics for ResNet50V2 model: Test Set-1 vs Test Set-2 (seed A).

### Comparing CNN architectures and robustness to data splits

In the first experiment, we evaluated the chosen CNN architectures (MobileNetV2, EfficientNetV2B0, and ResNet50V2) on pre-processed segmented images of size $256 \times 256$. The test set used for this experiment was Test Set-1 (time point 2208 in Fig. 3, and Table 1). Transfer learning was applied to all models, utilizing weights pretrained on ImageNet.

To investigate the robustness of the model and its sensitivity to the order in which images are presented during training, we tested three different randomly generated initialization seeds A, B and C. Comparing performance using different seeds allows us to assess the model’s consistency across different training and validation splits. It also gives clues about potential biases caused by fixed data divisions and in turn whether there are enough examples of the different classes. The seeds were kept fixed throughout all experiments to ensure reproducibility and eliminate bias from specific data shuffling or weight initialization. The actual seed values are not critical for interpretation, as their role is purely to validate stability across independent training runs.

As summarized in Table 3 and detailed in Table 2, ResNet50V2 and EfficientNetV2B0 both show good and stable performance (test accuracy and balanced test accuracy) across the different seeds, with EfficientNetV2B0 performing somewhat better than ResNet50V2. MobileNetV2 performs slightly worse and varies more between seeds. The training accuracy is higher, but still relatively close to the validation and test accuracy. Similar performance is expected for large enough and representative datasets. An illustration of the per class performance for the 3 models (seed A) is shown in Fig. 4 . There, it is clearly seen that it is the classes with a smaller number of samples for which the performance is not as good and varies the most. A closer look at the confusion matrices (Supplementary, Table S1), and interactive visual inspection of outlier and misclassification images in a UMAP projection (Supplementary Figure S1), reveal that misclassification is often due to multiclass characteristics or dubious annotation, most clearly so for moldy kernels.

To test whether misclassifications stemmed from Test Set-1’s characteristics or from general CNN limitations, we also evaluated performance on a different test set, Test Set-2. This also allowed us to better understand the model’s ability to generalize. For this investigation we chose ResNet50V2.

**Table 3:** Summary of model performance across the three seeds (mean ± std).

Model	Train Acc (%)	Val Acc (%)	Test Acc (Bal Acc) (%)
MobileNetV2 (Test-set 1)	95.2 ± 2.1	90.5 ± 4.8	91.7 ± 3.9 (88.9 ± 1.9)
EfficientNetV2B0 (Test-set 1)	96.5 ± 0.3	94.6 ± 0.4	96.2 ± 0.4 (92.5 ± 0.3)
ResNet50V2 (Test-set 1)	98.1 ± 0.2	94.9 ± 0.5	96.8 ± 0.5 (91.7 ± 0.6)

**Table 4:** Impact of pre-processing techniques and downscaling on per-class Recall.

Class	Segmented	Raw-Resized		Raw
	256$\times$256	256$\times$256	512$\times$512	792$\times$830
Sound	0.98	0.97	0.94	0.99
Insect	0.96	0.86	0.86	0.89
Moldy	0.72	0.65	0.74	0.77
Spotted	0.90	0.70	0.79	0.82
Fusarium	0.96	0.89	0.95	0.97
Black Germ	0.82	0.88	0.88	0.90
Sprouted	0.93	0.75	0.90	0.92
Broken	0.99	0.96	0.99	0.99
**Average recall (balanced accuracy)**	**0.91**	**0.83**	**0.88**	**0.91**

Next, we therefore trained a model on a different training, validation and test dataset division (Test Set-2, as described in the previous section), with pre-processed segmented images and using Seed A for the random splits. As before, we fine-tuned the ImageNet pretrained model on our dataset. The classification performance is shown in the last row of Table 2. The results show lower performance on Test Set-2 compared to Test Set-1. The test accuracy is 90.6%, compared to 96.3% for Test Set-1. The per-class performance (precision, recall and F1-score) is compared to Test Set-1 in the line chart in Fig. 5. The Y-axis represents the score ranging from 0 to 1, while the X-axis lists the wheat classes.

We observe that lower performance on Test Set-2 depends on higher misclassification rates for the classes “Moldy” and “Black Germ”. Precision and recall values indicate that many false positives are found for these classes, while simultaneously “Spotted” and “Sprouted” seem to miss some true instances. Additionally, “Insect” also shows noticeable higher errors for Test Set-2 with both more missed instances and false positives. This is confirmed by a closer analysis of the confusion matrix (Supplementary Table S2 ). These results indicate some limiting factors in the classification set-up, such as representativeness of the data, and likely incorrect/questionable annotations. This was visually confirmed via interactive UMAP visualization of misclassified images. Some classes in Test Set-2 seem to contain some characteristics that were not represented in the training data.

### Impact of pre-processing and resolution on classification performance

We hypothesize that pre-processing the images might be unnecessary for CNN based classification. We also hypothesize that some classes might require higher resolution images to capture fine class specific details.

To investigate this, we trained ResNet50V2 (using seed A) on raw, unsegmented images ($792 \times 830$ and the raw images downscaled to different sizes ($512 \times 512$ and $256 \times 256$) . We selected Test Set-1 for the experiment as it provided stable and consistent results in experiment 1. This decision was not about achieving the highest accuracy but ensuring reliable evaluation in this experiment.

The results presented in Table 4, together with the precision and recall heatmaps in Supplementary Figure S3, show that Pre-processed images, (size $256 \times 256$) achieved the highest average recall of 0.91, performing well for distinct classes such as *Sound* (0.98) and *Broken* (0.99). However, raw images downscaled to the same size ($256 \times 256$) showed a decrease in average recall to 0.83, with challenging classes like *Spotted* (0.70) and *Sprouted* (0.75) being severely affected. Downscaling only to $512 \times 512$ preserves finer details, which improves the average recall somewhat to 0.88. The original, high resolution raw images of size $792 \times 830$, has a similar performance as the pre-processed downscaled images with an average recall of 0.91. The original full size images however have higher recall for certain classes *Black Germ* (0.90) and *Moldy* (0.77), while pre-processing seems beneficial for especially *Insect* and *Spotted*. However, the comparison for these classes and especially Black Germ and Moldy on precision shows the opposite. That is, precision for Black Germ and Moldy is lower for the origi-nal full size images compared to the pre-processed downscaled images. This means that more are classified as Moldy and Black Germ for the original full size images including both true and false samples. It thus seems as if the pre-processed and original full sized images provide complementary information. To further validate the performance and robustness for the raw full sized images, we trained models using random seeds B and C (as in the first experiment). The results confirm similar and stable performance for all three random seeds. Confusion matrices for this experiment are provided in Supplementary Table S3.

### Implementation details

All models were trained and evaluated on a high-performance computing server equipped with four NVIDIA TITAN Xp GPUs (12 GB each), running CUDA Version 12.3 and NVIDIA-SMI Driver Version 545.29.06. The models were developed using TensorFlow 2.10 and Python 3.9.

Each model was trained for 50 epochs, with a batch size of 32 on a single GPU. A learning rate scheduler^28^ was implemented to optimize convergence, starting with an initial learning rate of $8 \times 10^{-4}$, which was reduced by 50% every 20 epochs according to a fixed schedule. Additionally, a dropout rate of 0.5 was applied to prevent overfitting. We used categorical cross-entropy^29^ as the loss function, as it is well-suited for multi-class classification tasks. Additionally, class imbalance was addressed by incorporating class weighting in the loss computation^30^. The class weights were calculated as the inverse of class frequencies, which ensures that minority classes contribute proportionally to the loss computation in training. The data was augmented using only random horizontal and vertical flips to avoid introducing additional resampling effects.

In contrast, for raw $792 \times 830$ images, during the initial training runs, validation accuracy fluctuated and plateaued after the 10th epoch, indicating that the learning rate and batch size of 8 were not optimal. To address this, the learning rate was reduced by 20% every 10 epochs to facilitate finer weight updates. Since the single GPU strategy was unable to handle the high batch size for full-resolution images, a Multi-GPU strategy was implemented. This enabled the batch size to be increased to 32, ensuring stable training and more efficient processing of high-resolution raw images ($792 \times 830$).

## Discussion

The results from the two experiments with different models, random seeds, pre-processing techniques, and different resolutions for wheat kernel images revealed key considerations for efficient and reliable real-world deployment.

Regarding the comparison of architectures and data splits, the results in Table 2 and the spider chart in Fig. 4 show a similar pattern for all three models investigated. The classes with more examples display better performance, while the smaller classes *Fusarium, Moldy, Spotted, and Black Germ* remain the most challenging for all models. Thus, it seems as if the number of examples and/or their representativeness are limiting factors for those classes. This is confirmed in the second part of the experiment, where the performance is compared for two different training-test set divisions. Those results also show that the trained model struggles with several of the smaller classes. A manual inspection of misclassified samples indicates that a non-negligible portion of errors can be attributed to ambiguous labeling and visually overlapping defect categories, where grains exhibit characteristics of more than one class. Representative examples of such ambiguous cases are shown in Supplementary Figure S2. In addition, the results indicate that the data time point used as the held out Test Set-2 has characteristics that differ from the data in the training set. Thus, confirming limitations regarding data size and representativeness. One common approach to address this is to apply advanced augmentation techniques (e.g., color, intensity, CutMix) to improve the size and representativeness of underrepresented classes. However, preliminary tests do not indicate improved performance in this application. This was therefore not explored further since we also did not find that so surprising due to that the built-in pre-processing includes intensity corrections and normalization, see Fig. 2. Mixing images might for this application mean that fine details necessary for a specific class are removed (and added to another class) and this technique is hence not appropriate for this application.

In the $2^{nd}$ experiment, the overall results (balanced accuracy) is the same for the pre-processed images of size 256 $\times$ 256 as for the original raw images of size 792 $\times$ 830, but the per class performance differs somewhat, see Table 4. This confirms our hypothesis that some classes benefit from the built-in pre-processing, whereas recognition of other classes benefit from the details found in the original full size images. The downsizing included in the pre-processing roughly corresponds to the raw images downscaled to 512 $\times$ 512. By comparing those results with the pre-processed, it is even more clearly seen that the pre-processing is beneficial for most classes. Comparing the raw downscaled with the raw full resolution, it is also clear that higher resolution increases the performance.

Taken together, the results suggest that addressing the shortcomings of existing algorithms requires combining robust CNN architectures with carefully designed pre-processing and evaluation pipelines. In particular, leveraging complementary information from both pre-processed and raw images may help balance fine-grained defect details with more generalizable representations. One plausible direction is multi-model fusion, where information from different networks or branches could be combined at an intermediate/late feature level or at the output level (combining the predictions from multiple independently trained networks), or weighted dynamically using attention-based mechanisms. Such approaches may improve robustness for visually challenging or ambiguous cases.

Finally, computational limitations during model training and at real-time inference within the instrument must be considered when designing and choosing solutions for practical deployment. This would be natural in the next step, i.e., when choosing mitigation strategies following identifying factors influencing performance (the focus of this paper).

## Conclusion

This study explores the performance of deep learning models in classifying images of individual wheat grains, focusing on model architecture, robustness, pre-processing and image resolution. Among the three architectures evaluated, ResNet50V2 and EfficientNetV2B0 showed similar and stable performance, and were slightly better than MobileNetV2. The per-class results indicate that the smaller classes suffer from lack of representative examples. However, interactive visualization of the data points in the UMAP projection reveal that another contributing factor is dubious annotation and multi-class belongingness. Either removing dubious or multi-label examples or updating the annotations and training and testing in multi-label setting would likely improve the results. It should be kept in mind though that when summarizing grain quality for a whole sample, only one class assigned to each kernel is preferred. Finally, we show that the built-in pre-processing has a large overall positive effect on performance, but that some classes benefit from higher resolution. Another way to improve performance would hence be to elucidate precisely what steps of the pre-processing that are important and combine only those with the full resolution images.

We can thus conclude that our systematic comparison has provided insights into what key factors influence classification performance in this wheat grain classification task. We can also conclude that those insights indicate paths to investigate for further improvement.

## Supplementary Information

Supplementary Information.

## Data Availability

A publicly available subset of the segmented wheat grain images used in this study has been deposited in Zenodo to support transparency and reproducibility. The dataset includes representative samples per class collected from instrument and can be accessed at https://doi.org/10.5281/zenodo.17397123. The full dataset is proprietary and cannot be shared due to confidentiality agreements with CGrain AB, but may be made available from the corresponding author upon reasonable request and subject to approval.

## Code availability

The models were implemented using TensorFlow and Python, with standard architectures (ResNet50V2, EfficientNetV2B0, MobileNetV2). No custom code beyond standard libraries was developed. Code to reproduce the training and evaluation pipeline can be shared upon request.

## References

[1] United States Department of Agriculture Foreign Agricultural Service. Production, Supply and Distribution Online (PS&D); accessed 19 Jan 2025. https://www.fas.usda.gov/data/production/commodity/0410000 (2025).

[2] United Nations. Global issues: Population; accessed 19 Jan 2025. https://www.un.org/en/global-issues/population (2025).

[3] Komyshev, E., Genaev, M. & Afonnikov, D. Evaluation of the seed counter, a mobile application for grain phenotyping. *Front. Plant Sci.* **7**, 1990. 10.3389/fpls.2016.01990 (2017). 28101093 10.3389/fpls.2016.01990 PMC5209368

[4] Elmasry, G., Mandour, N., Al-Rejaie, S., Belin, E. & Rousseau, D. Recent applications of multispectral imaging in seed phenotyping and quality monitoring-an overview. *Sensors* **19**(5), 1090. 10.3390/s19051090 (2019). 30836613 10.3390/s19051090 PMC6427362

[5] Cgrain. Innovating the future of agriculture with artificial intelligence; accessed 19 Jan 2025. https://www.cgrain.se (2025).

[6] Agarwal, D. et al. Machine learning approach for the classification of wheat grains. *Smart Agric. Technol.* **3**, 100136. 10.1016/j.atech.2022.100136 (2023).

[7] Unlersen, M. F. et al. CNN-SVM hybrid model for varietal classification of wheat based on bulk samples. *Eur. Food Res. Technol.* **248**(8), 2043–2052. 10.1007/s00217-022-04029-4 (2022).

[8] Velesaca, H. O., Suárez, P. L., Mira, R. & Sappa, A. D. Computer vision based food grain classification: A comprehensive survey. *Comput. Electron. Agric.* **187**, 106287. 10.1016/j.compag.2021.106287 (2021).

[9] Lingwal, S., Bhatia, K. K. & Tomer, M. S. Image-based wheat grain classification using convolutional neural network. *Multimed. Tools Appl.* **80**(28), 35441–35465. 10.1007/s11042-020-10174-3 (2021).

[10] Chen, F. et al. Classification of wheat grain varieties using terahertz spectroscopy and convolutional neural network. *J. Food Compos. Anal.* **129**, 106060. 10.1016/j.jfca.2024.106060 (2024).

[11] Yasar, A., Golcuk, A. & Sari, O. F. Classification of bread wheat varieties with a combination of deep learning approach. *Eur. Food Res. Technol.* **250**(1), 181–189. 10.1007/s00217-023-04375-x (2024).

[12] Yasar, A. Benchmarking analysis of CNN models for bread wheat varieties. *Eur. Food Res. Technol.* **249**(3), 749–758. 10.1007/s00217-022-04172-y (2023).

[13] Hossen, M. H. et al. Wheat diseases detection and classification using convolutional neural network (CNN). *Int. J. Adv. Comput. Sci. Appl.* (2022). 10.14569/IJACSA.2022.0131183

[14] Nguyen, T. T. et al. Monitoring agriculture areas with satellite images and deep learning. *Appl. Soft Comput.* **95**, 106565. 10.1016/j.asoc.2020.106565 (2020).

[15] Wang, C. et al. Deep learning based high-throughput phenotyping of chalkiness in rice exposed to high night temperature. *Plant Methods* **18**(1), 9. 10.1186/s13007-022-00839-5 (2022). 35065667 10.1186/s13007-022-00839-5 PMC8783510

[16] Ali, W., Din, I. U., Almogren, A. & Rodrigues, J. J. Poultry health monitoring with advanced imaging: Towards next-generation agricultural applications in consumer electronics. *IEEE Trans. Consum. Electron.*10.1109/TCE.2024.3409069 (2024).

[17] Yang, M. et al. Insect recognition method with strong anti-interference capability for next-generation consumer imaging technology. *IEEE Trans. Consum. Electron.*10.1109/TCE.2024.3411567 (2024).

[18] Fernández-Campos, M. et al. Wheat spike blast image classification using deep convolutional neural networks. *Front. Plant Sci.* **12**, 673505. 10.3389/fpls.2021.673505 (2021). 34220894 10.3389/fpls.2021.673505 PMC8248543

[19] Ceyhan, M., Kartal, Y., Özkan, K. & Seke, E. Classification of wheat varieties with image-based deep learning. *Multimed. Tools Appl.* **83**(4), 9597–9619. 10.1007/s11042-023-16075-5 (2024).

[20] Yasar, A. Analysis of selected deep features with CNN-SVM-based for bread wheat seed classification. *Eur. Food Res. Technol.* **250**(6), 1551–1561. 10.1007/s00217-024-04488-x (2024).

[21] Kozłowski, M. et al. Identifying defects and varieties of malting barley kernels. *Sci. Rep.* **14**(1), 22143. 10.1038/s41598-024-73683-3 (2024). 39333255 10.1038/s41598-024-73683-3 PMC11436987

[22] Sandler, M., Howard, A., Zhu, M., Zhmoginov, A., & Chen, L.-C. Mobilenetv2: Inverted residuals and linear bottlenecks. In *Proceedings of the IEEE Conference on Computer Vision and Pattern Recognition* 4510–4520 (2018). 10.1109/CVPR.2018.00474.

[23] Tan, M., & Le, Q. Efficientnetv2: Smaller models and faster training. In *International Conference on Machine Learning* 10096–10106 (PMLR, 2021). 10.48550/arXiv.2104.00298.

[24] He, K., Zhang, X., Ren, S., & Sun, J. Deep residual learning for image recognition. In *Proceedings of the IEEE Conference on Computer Vision and Pattern Recognition* 770–778 (2016). 10.1109/CVPR.2016.90.

[25] Sokolova, M. & Lapalme, G. A systematic analysis of performance measures for classification tasks. *Inf. Process. Manag.* **45**(4), 427–437. 10.1016/j.ipm.2009.03.002 (2009).

[26] Brodersen, K. H., Ong, C. S., Stephan, K. E., & Buhmann, J. M. The balanced accuracy and its posterior distribution. In *2010 20th International Conference on Pattern Recognition* 3121–3124 (IEEE, 2010). 10.1109/ICPR.2010.764.

[27] Manning, C.D., Raghavan, P., & Schütze, H. Boolean retrieval. In *Introduction to information retrieval* 1–18 (2008). 10.1017/CBO9780511809071.

[28] Smith, L. N. Cyclical learning rates for training neural networks. In *2017 IEEE Winter Conference on Applications of Computer Vision (WACV)* 464–472 (IEEE, 2017). 10.1109/wacv.2017.58.

[29] Goodfellow, I., Bengio, Y., Courville, A., & Bengio, Y. *Deep learning* (MIT Press, 2016). 10.4258/hir.2016.22.4.351

[30] Sze-To, A., & Wong, A. K. A weight-selection strategy on training deep neural networks for imbalanced classification. In *Image Analysis and Recognition: 14th International Conference, ICIAR 2017, Montreal, QC, Canada, July 5–7, 2017, Proceedings 14* 3–10 (Springer, 2017). 10.1007/978-3-319-59876-5_1.

