# Supplementary material for: Investigating performance and key factors for real-world deployment of grain image classification using convolutional neural networks
Source: Sci Rep. 2026 Apr 14;16:12357. doi: 10.1038/s41598-026-45314-6 (PMC13079808; doi:10.1038/s41598-026-45314-6)
Supplement: Supplementary file 1 — Supplementary Information. [file 41598_2026_45314_MOESM1_ESM.pdf]

Supplementary information.

Supplementary Information

This supplementary section includes additional analyses and visualizations to complement the findings discussed in the main text. Extended results, such as precision and recall heatmaps, UMAP visualization, confusion matrices, and further discussion on model performance across different image resolutions, are provided.

S1. UMAP Visualization

To better understand the feature embeddings learned by the ResNet50V2 model on segmented and downscaled wheat grain images, UMAP was utilized for dimensionality reduction. The UMAP plot in Supplementary Figure S1 provides a visual representation of how the model distinguishes between different classes in the feature space. UMAP was fitted to the training data embeddings and used to project the test set into the same feature space, ensuring consistency between the training and test distributions.

The visualizations reveal well-separated clusters for most classes, indicating effective feature learning and generalization. However, certain classes, particularly *Moldy* and *Black Germ*, exhibit significant overlap and dispersion, which explains the higher misclassification rates observed for these categories.

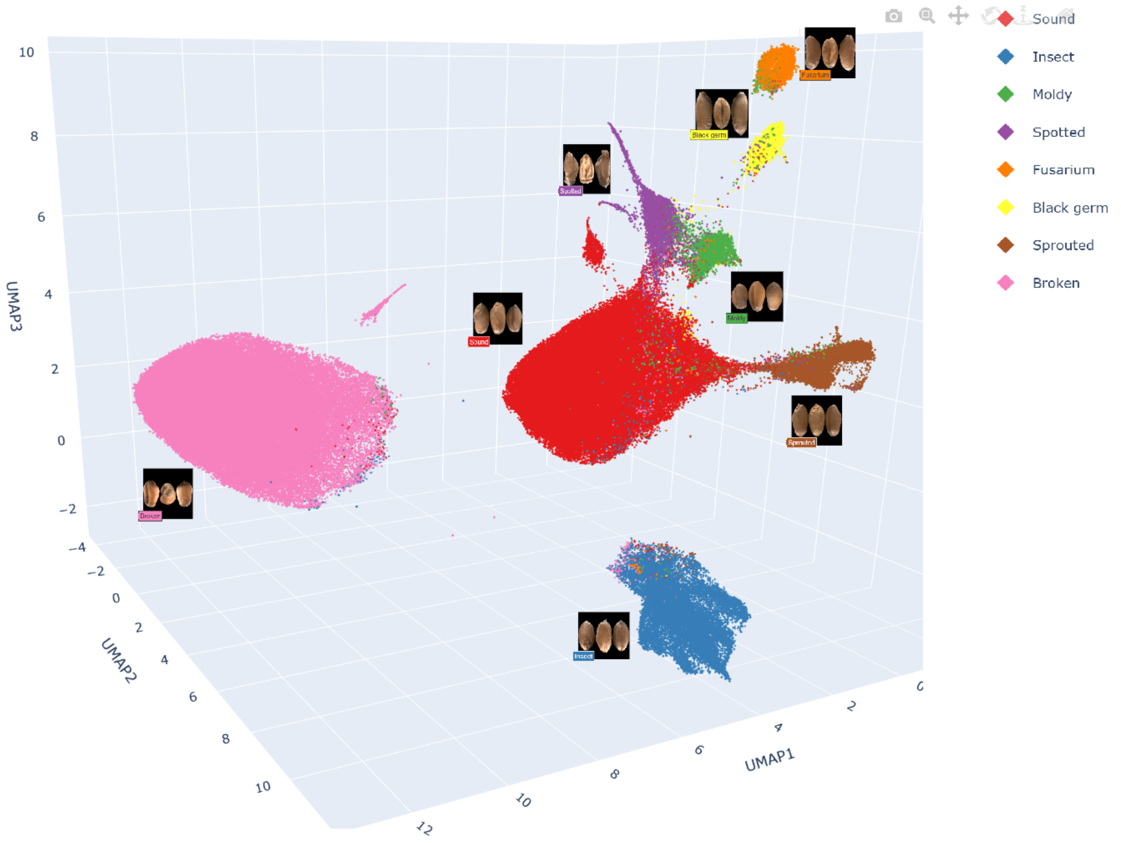

Fig. S1: UMAP visualization of feature embeddings from the ResNet50V2 model on segmented images. Different colors represent different classes in the dataset.

- **Moldy**: The *Moldy* class shows a scattered distribution in the UMAP space, with substantial overlap with visually similar classes like *Spotted* and *Fusarium*. This overlap suggests that the model struggles to extract distinct features for *Moldy*, leading to frequent misclassifications.
- **Black Germ**: The *Black Germ* class forms smaller and fragmented clusters, with considerable overlap into the *Spotted*, *Sound* and *Moldy* regions. The dispersed nature of these embeddings indicates that the model has difficulty capturing unique characteristics for *Black Germ*, contributing to its misclassification.

The UMAP visualization thus highlights the challenging nature of these classes due to their visual similarities with other categories. These insights suggest the need for further refinement in feature learning, possibly through targeted data augmentation, specialized pre-processing, or improved model architectures to better differentiate these problematic classes.

S2. Analysis of Annotation Ambiguity

Visual inspection of misclassified samples (class outliers in the UMAP projection shown in Supplementary Figure S1) indicates that some of the errors can be attributed to ambiguous labeling and visually overlapping defect categories, where grains exhibit characteristics of more than one class. A few examples of such ambiguous cases are shown in Supplementary Figure S2. The extent of this problem was not further investigated in this study.

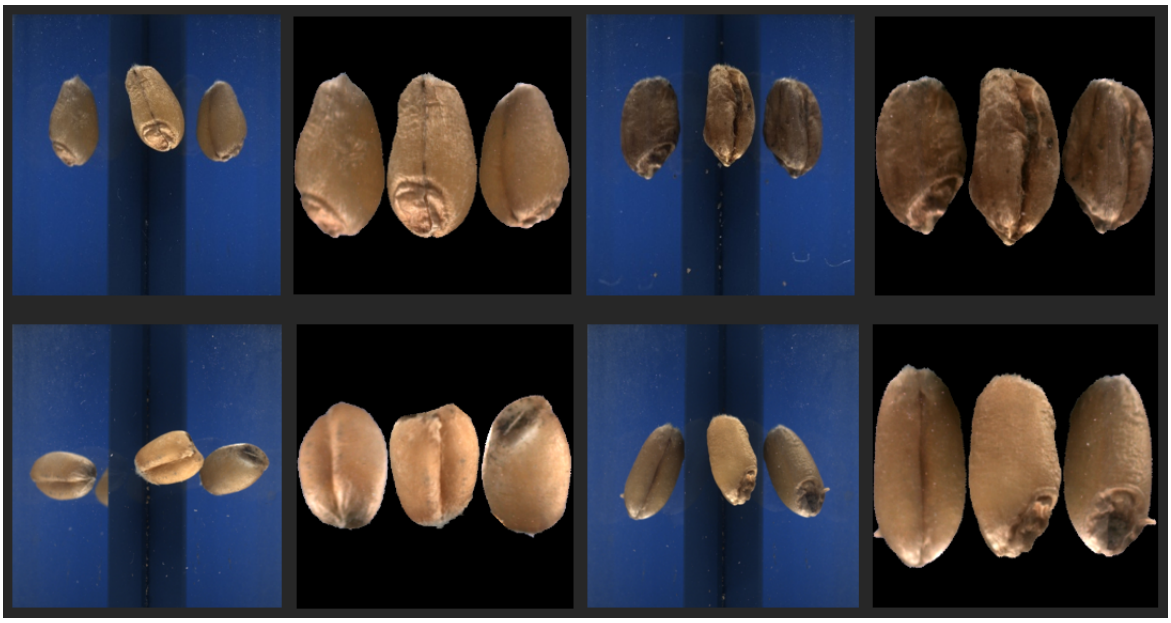

Fig. S2: Representative examples of wheat grains with dubious annotation and/or ambiguous or multi-class visual characteristics. The first row shows paired original and segmented images of (left) a kernel erroneously annotated as Broken despite being Sound, and (right) a kernel annotated as Moldy that displays Fusarium characteristics. The second row shows (left) a kernel annotated as Insect-damaged that (also) looks Broken, and (right) a kernel annotated as Sprouted that looks Moldy.

S3. Precision and Recall Heatmap

The heatmap in Supplementary Figure S3 illustrates the precision and recall performance of the ResNet50V2 model (seed A) across the preprocessed and segmented images and raw images downscaled to different sizes. The color scale ranges from light to dark, where darker shades (values close to 1.0) represent higher precision/recall, and lighter shades (values closer to 0.6) indicate lower performance.The model performs well across all resolutions, with most classes having precision and recall values above 0.85.

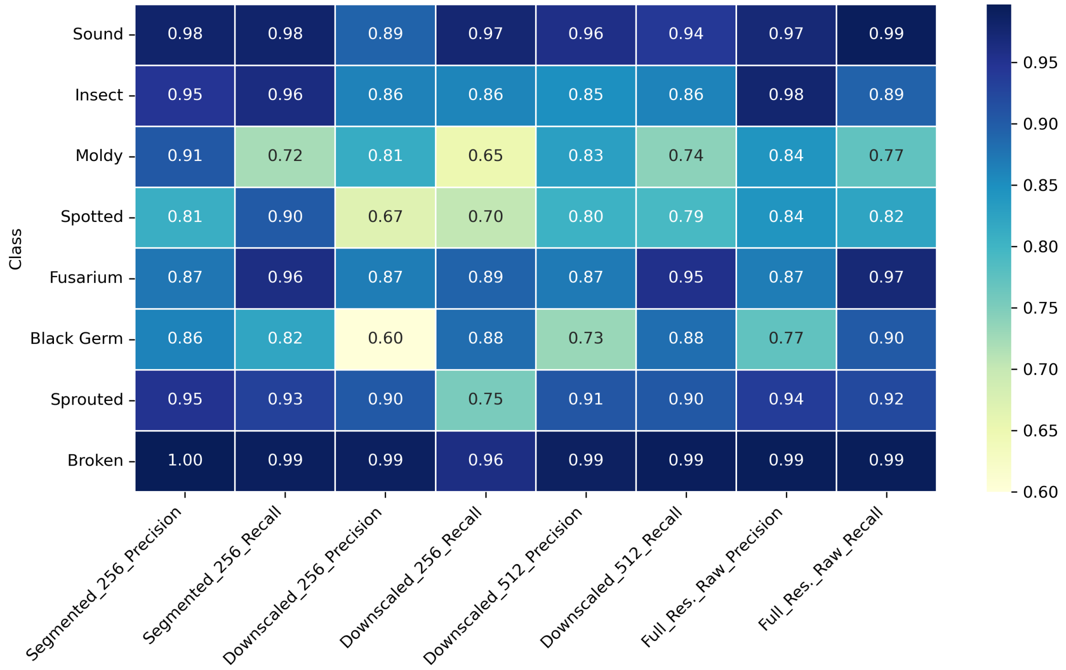

Fig. S3: Precision and Recall heatmap for ResNet50V2 for the preprocessed and segmented images (2 left-most columns) and raw full size images (2 right-most columns) and raw downscaled to different sizes (4 middle columns).

The *Broken* class achieves the highest performance, reaching 0.99 for both metrics. The high-resolution raw images (792×830) improved classification for challenging classes *Moldy* and *Black Germ*. Conversely, downscaling to 256×256 slightly reduced performance for subtle classes like *Spotted*, *Sprouted*, and *Moldy*, indicating areas for potential improvement.

S4. Confusion Matrices

Supplementary Tables S1 to S3 present the confusion matrices for the evaluated models, providing detailed insights into class-wise performance and misclassification patterns. Supplementary Table S1 shows the confusion matrices for MobileNetV2, EfficientNetV2B0, and ResNet50V2 evaluated on Test Set-1 and trained using seed A. The matrices highlight correct predictions along the diagonal (marked in bold blue), while significant misclassifications (errors exceeding 100 samples) are indicated in red. These matrices offer a comparative overview of how each model handles various wheat grain classes and where misclassifications commonly occur. Supplementary Table S2 displays the confusion matrix for ResNet50V2 evaluated on Test Set-2, and trained using seed A. This table highlights a notable decline in the classification performance for certain classes such as *Moldy* and *Sprouted*, revealing challenges in generalization when tested on a different dataset split. Supplementary Table S3 presents confusion matrices comparing ResNet50V2's performance on downscaled images (256×256, 512×512) and raw images (792×830) on Test-Set-1 and trained with Seed A. The results indicate that higher-resolution images generally enhance classification, especially for visually subtle classes like *Black Germ* and *Moldy*. The downscaled images exhibit more misclassifications, particularly among classes with fine-grained details. The bottom part of the table shows confusion matrices for ResNet50V2 on Test-Set 1 for the raw, full resolution images trained with different random seeds (Seed A, B, and C). This analysis explores the impact of data splits on model performance and highlights consistency or variability across different training runs. These confusion matrices serve to complement the precision, recall, and F1-score analyses in the main text, offering a granular view of where models succeed and where they struggle.

Table S1: Comparison of Confusion Matrices for MobileNetV2, EfficientNetV2B0, and ResNet50V2 (Seed A) on Test Set-1, pre-processed and segmented images

**MobileNetV2**								
**Actual \Predicted**	**Sound**	**Insect**	**Moldy**	**Spotted**	**Fusarium**	**Black Germ**	**Sprouted**	**Broken**
**Sound**	**8898**	13	8	11	6	7	5	1
**Insect**	127	**2681**	2	8	3	4	12	13
**Moldy**	38	11	**1606**	193	202	126	18	2
**Spotted**	42	2	77	**1644**	4	41	16	1
**Fusarium**	3	2	44	16	**1655**	1	4	0
**Black Germ**	13	0	6	19	0	**448**	0	0
**Sprouted**	36	14	39	40	1	40	**2180**	1
**Broken**	39	63	25	31	7	3	6	**16585**
**EfficientNetV2B0**								
**Actual \Predicted**	**Sound**	**Insect**	**Moldy**	**Spotted**	**Fusarium**	**Black Germ**	**Sprouted**	**Broken**
**Sound**	**8809**	28	51	30	11	2	14	4
**Insect**	48	**2754**	7	5	2	2	14	18
**Moldy**	8	2	**1794**	111	215	41	23	2
**Spotted**	12	2	124	**1631**	5	29	20	4
**Fusarium**	0	12	31	10	**1666**	1	3	2
**Black Germ**	6	0	34	16	0	**430**	0	0
**Sprouted**	12	14	53	35	2	13	**2212**	10
**Broken**	5	46	8	18	6	2	5	**16669**
**ResNet50V2**								
**Actual \Predicted**	**Sound**	**Insect**	**Moldy**	**Spotted**	**Fusarium**	**Black Germ**	**Sprouted**	**Broken**
**Sound**	**8827**	47	16	39	8	1	9	2
**Insect**	53	**2748**	3	5	1	3	14	23
**Moldy**	32	19	**1600**	239	221	27	50	8
**Spotted**	26	3	71	**1662**	7	29	25	4
**Fusarium**	6	16	20	4	**1665**	1	7	6
**Black Germ**	12	0	26	42	0	**402**	4	0
**Sprouted**	36	19	28	50	2	4	**2206**	6
**Broken**	1	51	4	14	3	0	7	**16679**

Table S2: Confusion Matrix for ResNet50V2 (Seed A) on Test Set-2, pre-processed and segmented images

**Actual \Predicted**	**Sound**	**Insect**	**Moldy**	**Spotted**	**Fusarium**	**Black Germ**	**Sprouted**	**Broken**
**Sound**	**11538**	33	131	23	30	5	43	4
**Insect**	78	**547**	97	13	1	4	17	8
**Moldy**	18	2	**496**	30	30	107	26	1
**Spotted**	98	6	219	**910**	26	84	75	4
**Fusarium**	8	7	54	4	**941**	3	10	1
**Black Germ**	0	0	7	5	0	**221**	7	0
**Sprouted**	555	46	116	27	14	19	**3083**	5
**Broken**	88	177	58	62	8	2	44	**6868**

Table S3: Comparison of Confusion Matrices for ResNet50V2 on Test Set-1, raw downscaled and full size images

256 × 256 Downscaled Images (Seed A)								
**Actual \Predicted**	**Sound**	**Insect**	**Moldy**	**Spotted**	**Fusarium**	**Black Germ**	**Sprouted**	**Broken**
**Sound**	**8720**	120	15	50	7	7	23	7
**Insect**	283	**2452**	3	10	1	6	14	81
**Moldy**	68	15	**1445**	359	179	79	41	10
**Spotted**	139	48	133	**1284**	13	109	76	25
**Fusarium**	13	37	58	30	**1552**	6	9	20
**Black Germ**	3	0	24	29	0	**429**	1	0
**Sprouted**	262	52	87	100	6	50	**1777**	16
**Broken**	288	117	16	57	31	29	25	**16196**

512 × 512 Downscaled Images (Seed A)								
**Actual \Predicted**	**Sound**	**Insect**	**Moldy**	**Spotted**	**Fusarium**	**Black Germ**	**Sprouted**	**Broken**
**Sound**	**8425**	362	50	34	9	4	38	27
**Insect**	199	**2478**	3	12	8	4	39	107
**Moldy**	19	13	**1626**	242	197	37	48	14
**Spotted**	43	2	139	**1449**	12	80	75	27
**Fusarium**	2	9	45	9	**1647**	2	5	6
**Black Germ**	1	0	32	19	0	**432**	2	0
**Sprouted**	76	28	58	25	4	21	**2125**	13
**Broken**	32	25	10	11	13	11	11	**16646**

792 × 830 Raw Images (Seed A)								
**Actual \Predicted**	**Sound**	**Insect**	**Moldy**	**Spotted**	**Fusarium**	**Black germ**	**sprouted**	**Broken**
**Sound**	**8883**	5	20	12	13	3	9	4
**Insect**	146	**2549**	11	29	11	3	22	79
**Moldy**	18	8	**1698**	188	210	33	37	4
**Spotted**	36	2	141	**1509**	7	65	62	5
**Fusarium**	1	5	29	9	**1675**	1	4	1
**Black germ**	2	0	34	7	0	**441**	1	1
**Sprouted**	47	20	69	25	1	17	**2165**	6
**Broken**	17	19	14	13	14	8	11	**16663**

792 × 830 Raw Images (Seed B)								
**Actual \Predicted**	**Sound**	**Insect**	**Moldy**	**Spotted**	**Fusarium**	**Black germ**	**sprouted**	**Broken**
**Sound**	**8893**	6	16	11	8	3	10	2
**Insect**	137	**2634**	3	9	2	1	14	50
**Moldy**	26	8	**1717**	178	185	37	38	7
**Spotted**	63	3	133	**1484**	9	74	50	11
**Fusarium**	2	5	46	6	**1659**	1	4	2
**Black germ**	1	0	34	11	0	**439**	1	0
**Sprouted**	68	18	77	14	2	19	**2145**	7
**Broken**	17	14	8	7	13	8	5	**16687**

792 × 830 Raw Images (Seed C)								
**Actual \Predicted**	**Sound**	**Insect**	**Moldy**	**Spotted**	**Fusarium**	**Black germ**	**sprouted**	**Broken**
**Sound**	**8892**	8	18	10	8	1	8	4
**Insect**	164	**2576**	6	37	3	3	11	50
**Moldy**	33	8	**1672**	213	197	33	34	6
**Spotted**	58	3	124	**1517**	7	69	46	3
**Fusarium**	2	3	36	4	**1676**	1	3	0
**Black germ**	1	0	27	12	0	**444**	2	0
**Sprouted**	122	26	75	34	1	20	**2065**	7
**Broken**	52	23	11	10	13	8	3	**16639**
